# Can Resveratrol Influence the Activity of 11β-Hydroxysteroid Dehydrogenase Type 1? A Combined In Silico and In Vivo Study

**DOI:** 10.3390/ph16020251

**Published:** 2023-02-07

**Authors:** Jurica Novak, Vadim E. Tseilikman, Olga B. Tseilikman, Svetlana S. Lazuko, Lyudmila E. Belyeva, Azam Rahmani, Julia Fedotova

**Affiliations:** 1 Department of Biotechnology, University of Rijeka, 51000 Rijeka, Croatia; 2 Center for Artificial Intelligence and Cyber Security, University of Rijeka, 51000 Rijeka, Croatia; 3 Scientific and Educational Center ‘Biomedical Technologies’, School of Medical Biology, South Ural State University, 454080 Chelyabinsk, Russia; 4 Faculty of Fundamental Medicine, Chelyabinsk State University, 454001 Chelyabinsk, Russia; 5 Department of Physiology, Vitebsk State Medical University, Frunze Av. 27, 210023 Vitebsk, Belarus; 6 Department of Pathophysiology, Vitebsk State Medical University, Frunze Av. 27, 210023 Vitebsk, Belarus; 7 Nursing and Midwifery Care Research Center, School of Nursing and Midwifery, Tehran University of Medical Sciences, Tehran P.O. Box 14665-354, Iran; 8 Laboratory of Neuroendocrinology, I.P. Pavlov Institute of Physiology RAS, 6 Emb. Makarova, 199034 Saint Petersburg, Russia

**Keywords:** 11β-hydroxysteroid dehydrogenase type 1, resveratrol, PTSD, corticosterone, molecular dynamics, plus maze test

## Abstract

The enzyme 11β-hydroxysteroid dehydrogenase type 1 (11β-HSD-1) is an NADPH-dependent reductase, responsible for the activation of cortisol by reducing cortisone. Resveratrol (RES), a type of natural polyphenol, is reported to be able to slow the progression of cancer and cardiovascular disease and improve the health of mice on a high-calorie diet. In this article, we applied molecular docking and molecular dynamics simulations to investigate the possibility of binding RES to 11β-HSD-1. The 11β-HSD-1:RES complex is stable on the μs time scale, and backbone RMSD-based clustering identified three conformations. Special attention was paid to the interaction pattern between the ligand and the target molecule, revealing hydrogen bonds between the hydroxyl group of RES and Thr124, as well as hydrophobic interactions responsible for the binding. In vivo studies demonstrated the ability of resveratrol at a dose of 40 mg/kg to reduce 11β-HSD-1 activity in the liver of rats under conditions of experimental post-traumatic stress disorder (PTSD), as well as in non-stressed animals. In both cases, the resveratrol-induced reduction in 11β-HSD-1 activity was accompanied by an increase in plasma corticosterone levels and a decrease in anxiety levels in the plus maze test.

## 1. Introduction

**Scheme 1:**
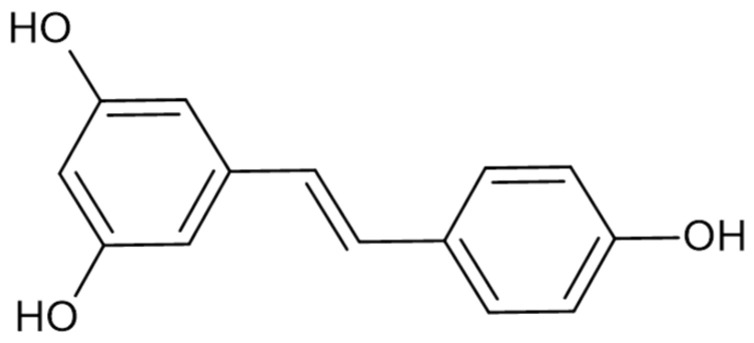
Two-dimensional structure of *trans*-resveratrol.

Resveratrol (RES, 3,5,4′-trihydroxy-*trans*-stilbene, Scheme 1), a natural polyphenol, belongs to the phytoalexins and is produced in the cells of many plants in response to stress. It is found in large quantities in red wine, the skins and seeds of grapes, and especially in the dried roots of plants [1].

The abundance of resveratrol’s beneficial biological effects is due to its multiple molecular targets, which give it the unique ability to modulate various intracellular signaling pathways on a micromolar scale [2]. In particular, by activating the SIRT1-PGC1α and AMPK signaling pathways, resveratrol has a protective effect on mitochondria [3]. The anti-inflammatory effect of resveratrol is associated with the modulation of the NF-κB signaling pathway [4]. The antioxidant effect is largely related to the activation of the PI3K/Akt-mediated Nrf2 pathway by resveratrol, ref. [5] and its antitumor effect is achieved through regulation of the PI3K/Akt/mTOR pathway [6]. Resveratrol modulates apoptosis, mediated by the Fas/Fas ligand, p53 [7]. Through its ability to regulate HIF-1α signaling, resveratrol improves cellular glucose metabolism [8]. The examples given here do not exhaust all the possibilities of resveratrol in regulating intracellular signaling pathways, but they demonstrate the possible mechanisms of protective action of resveratrol in various pathologies. Experimental and clinical studies have shown that resveratrol is able to correct various diseases of the cardiovascular system, [9] type 2 diabetes, [10] metabolic syndrome, [11] immune disorders [12], and oncopathology [13]. Resveratrol is also effective in certain neurodegenerative diseases such as Alzheimer’s disease [14] and chronic stress-induced behavioral disorders [15]. The protective effect of resveratrol in these pathologies is also related to its neuroprotective effect, which is manifested in increased neuroplasticity and increased levels of brain-derived neurotrophic factor (BDNF) and other neurotrophins in various brain structures [16]. Anxiety–depressive disorders are characterized by a decrease in BDNF expression in the prefrontal cortex and hippocampus [17]. Similar changes in BDNF have been observed in post-traumatic stress disorder (PTSD) [18]. In the context of PTSD, the involvement of hepatic 11β-HSD-1 in the pathogenesis of this disorder has been established [19].

Enzymes that belong to the 11-β-hydroxysteroid dehydrogenase (11β-HSD) family act predominantly at the local, tissue level [20], and 11β-HSD has two isoforms: 11β-HSD-1 and 11β-HSD-2. The former is located on the luminal surface of the endoplasmic reticulum and is dependent on nicotinamide adenine dinucleotide phosphate (NADPH) [21]. It is highly expressed in the brain, adrenal glands, liver, and adipocytes and converts inactive cortisone to cortisol [22]. Clinical data indicate increased activity of 11β-HSD-1 in patients with PTSD [23]. Data from experimental PTSD in rats shows an association between increased activity of 11β-HSD-1 in the liver and the severity of behavioral disturbances in PTSD [24]. This fact is in agreement with data from mice exposed to PTSD in which the 11β-HSD-1 gene was genetically knocked out and in which the signs of PTSD were less pronounced than in the wild type [25]. On the basis of the facts presented here, we hypothesized that the ability of resveratrol to correct behavioral responses might be related to its ability to regulate 11β-HSD-1 activity in the liver. This study is dedicated to testing this hypothesis.

## 2. Results

### 2.1. In Vivo Results

#### 2.1.1. Effects of *Trans*-Resveratrol Treatment on Hepatic 11β-HSD-1 Activity and Plasma Corticosterone Levels

**Figure 1:**
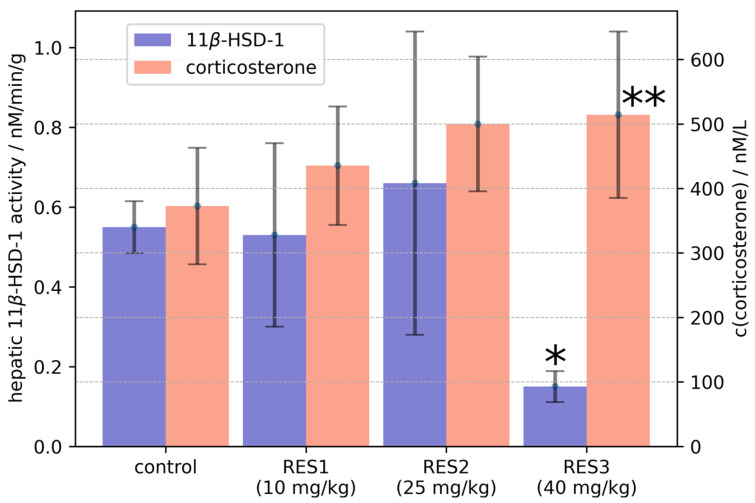
The influence of resveratrol treatment on hepatic 11β-HSD-1 activity and plasma corticosterone concentration. Graph is plotted as mean value ± SD. Statistical analysis included one-sided ANOVA. Significant values are expressed as * *p* < 0.0001, ** *p* < 0.01.

The statistical analysis showed a significant effect of RES dose (*p* < 0.005) on hepatic 11β-HSD-1 activity levels. A post hoc assay showed that rats treated with RES at a dose of 40 mg/kg had decreased 11β-HSD-1 enzyme activity (Figure 1), whereas treatment at a dose of 10 mg/kg as well as treatment at a dose of 25 mg/kg had no significant effect on the activity of this enzyme.

The one-sided ANOVA test showed a significant effect of RES dose (*p* < 0.05) on plasma corticosterone levels (Figure 1). The post hoc assay showed that treatment with RES at a dose of 40 mg/kg increased plasma corticosterone levels. The post hoc analysis showed no significant effects of the 10 mg/kg dose and the 25 mg/kg dose on plasma corticosterone concentration. In rats treated with RES at the 40 mg/kg dose, hepatic 11β-HSD-1 activity was negatively correlated with plasma corticosterone concentration (R = −0.867).

#### 2.1.2. Effects of *Trans*-Resveratrol Treatment on the Elevated plus Maze (EPM) Test Values

The effects of RES treatment on EPM test scores are shown in Table 1. Significant effects of RES dose on time spent in open arms (*p* < 0.0001), time spent in closed arms (*p* < 0.0001), the number of entries into closed arms (*p* < 0.001), and anxiety index (AI) (*p* < 0.0001) were observed. The post hoc study showed that treatment with RES at a dose of 40 mg/kg increased the time spent in the open arms, decreased the time spent in the closed arms, and decreased the number of entries into the closed arms and AI score. There were no significant effects of treatment with RES on EPM test scores at the 10 mg/kg dose and the 25 mg/kg dose. In rats treated with RES at a dose of 40 mg/kg, hepatic 11β-HSD-1 activity was positively correlated with AI scores (R = 0.9).

#### 2.1.3. Effects of *Trans*-Resveratrol Treatment on Hepatic 11β-HSD-1 Activity and Plasma Corticosterone Levels in Rats with PTSD

**Figure 2:**
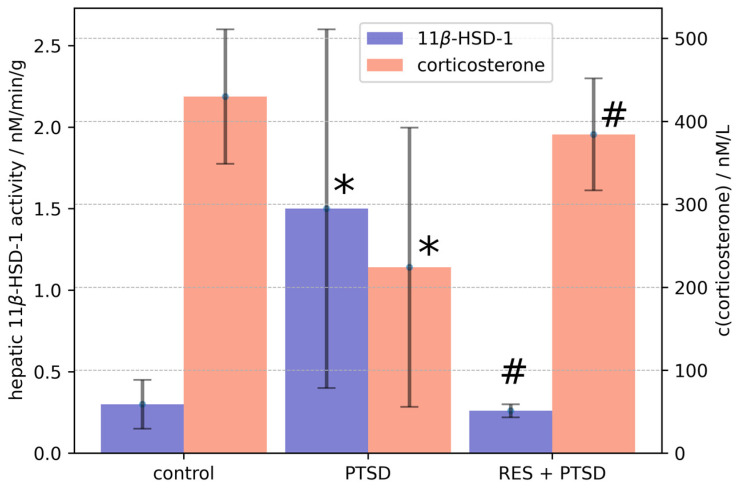
Effect of resveratrol treatment on PTSD-induced changes in hepatic 11β-HSD-1 activity and plasma corticosterone concentration. Graph is plotted as mean values ± SD. Statistical analysis included one-sided ANOVA. Significant values are expressed as * = significant difference with control, # = significant difference between PTSD and RES + PTSD groups.

The statistical analysis using a one-sided ANOVA showed the significant effect of PTSD on hepatic 11β-HSD-1 activity (*p* < 0.0001) and plasma corticosterone concentration (*p* < 0.005) (Figure 2). Hepatic 11β-HSD-1 activity was fivefold higher in PTSD rats than in control rats (Figure 2). The PTSD-induced increase in hepatic 11β-HSD-1 activity was blocked by *trans*-resveratrol treatment at 40 mg/kg. The plasma corticosterone concentration was reduced twofold in PTSD rats compared with the control group (Figure 2). Treatment of PTSD rats with *trans*-resveratrol at a dose of 40 mg/kg completely prevented the decrease in plasma corticosterone concentration. As a promising inhibitor targeting 11β-HSD-1, the IC_50_ of resveratrol was 22.75 ± 5.6 μM (22.75%).

#### 2.1.4. Effects of *Trans*-Resveratrol Treatment on EPM Test Scores in PTSD Rats

EPM test values are shown in Table 2. The one-sided ANOVA test showed significant effects of PTSD on time spent in open arms (*p* < 0.0001), time spent in closed arms (*p* < 0.0001), the number of entries into the closed arms (*p* < 0.001), and AI score (*p* < 0.0001). PTSD rats showed a significant decrease in time spent in the open arms and an increase in time spent in the closed arms. The number of entries into the closed arms and the AI score were also significantly increased. Treatment with trans-resveratrol at a dose of 40 mg/kg significantly increased the time spent in the open arms and decreased the time spent in the closed arms compared with these times for PTSD rats. Trans-resveratrol also caused a significant decrease in AI scores.

### 2.2. Molecular Docking and Molecular Dynamics Simulations

Being aware of the advantages and potential problems of molecular docking experiments [19], we performed docking of resveratrol to the 11β-HSD-1 receptor. The docking procedure was validated via re-docking of the SFF ligand to the 11β-HSD-1 binding site. The root mean squared deviation (RMSD) between the experimental and docked structure was low (0.18 Å), corroborating the correctness of our approach. Overlaid experimental and docked SFF structures in protein pockets are displayed in Figure S1.

**Figure 3:**
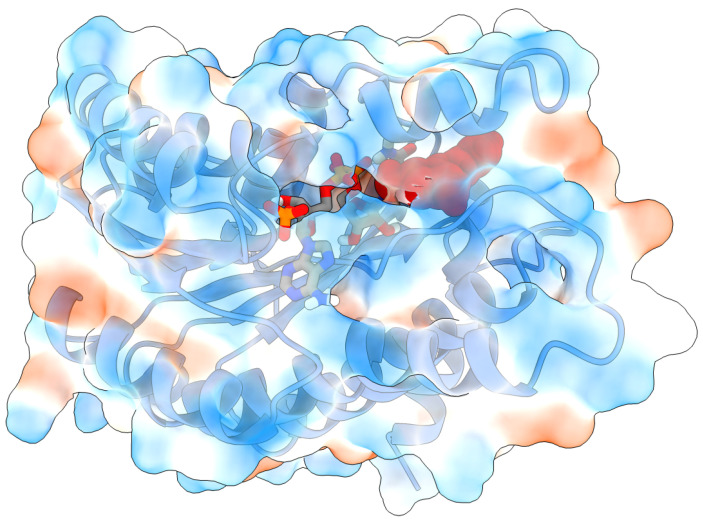
Initial structure of RES:11β-HSD-1 complex for molecular dynamics simulations obtained by docking, showing 11β-HSD-1 protein (PDB ID 4K1L), NADPH cofactor (licorice model), resveratrol (vdW model, red), and hydrophobic (blue surface) and hydrophilic (red surface) residues.

Based on the docking scoring function from AutoDock Vina, the free energy of binding of resveratrol to 11β-HSD-1 is −8.0 kcal/mol. The ligand is positioned in the vicinity of the carbamoylpyridin moiety of NADPH cofactor and is completely encircled by protein—mostly by hydrophobic residues (Figure 3). Since the stability of protein–ligand complexes in general depends on the molecular interactions and the solvent conditions around the protein and considering the fact that the protein was held fixed during the docking simulation, the described docked structure was taken as the initial geometry of the complex for MD simulation.

**Figure 4:**
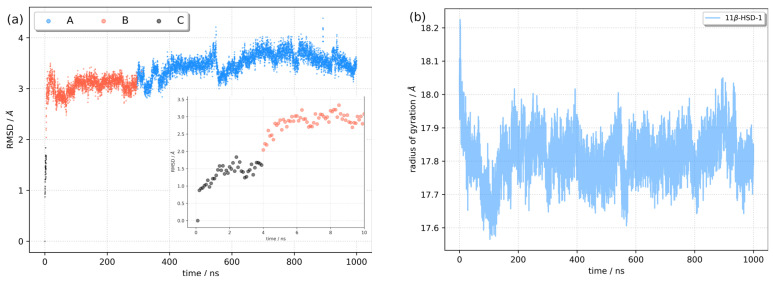
Stability of the RES:11β-HSD-1 complex during molecular dynamics simulation. (**a**) Root mean square deviation with position of the representative conformations shown (A, B, C, vide infra) and (**b**) radius of gyration.

With the aim of exploring the conformational dynamics and stability of the RES:11β-HSD-1 complex, a 1 μs long molecular dynamics simulation was performed. The root mean square deviation (RMSD) and radius of gyration (RoG) of the complex were calculated against the initial structure to monitor complex stability (Figure 4). The system reached equilibrium in 10–20 ns, with the average RMSD fluctuating around 3.36 ± 0.31 Å. The cluster analysis (vide infra) suggested a conformational change of the protein at around 4 ns and 300 ns. When the RMSD values were divided into two time periods (from the beginning of the simulation until 300 ns and from 300 ns until the end) and averages were calculated, it was observed that the RMSD for the first segment was 3.03 ± 0.25 Å, and that for the second was 3.50 ± 0.20 Å. The radius of gyration plotted against simulation time confirms the convergence of the trajectory and the stability of the complex. Being the measurement of the distribution of atoms in the complex, RoG is a valuable descriptor, depicting the compactness of the complex. The mean RoG value was 17.8 ± 0.3 Å, and splitting the RoG values into two segments (as for the RMSD) did not reveal significant differences.

**Figure 5:**
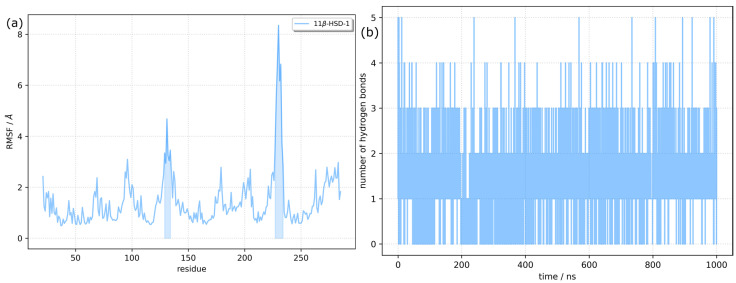
Trajectory analysis. (**a**) Root mean square fluctuations per residue of the RES:11β-HSD-1 complex. Regions with an RMSF above 3 Å are highlighted; (**b**) Number of hydrogen bond fluctuations.

The flexibility of different parts of the 1β-HSD-1 protein in the complex was investigated by calculation of the root mean square fluctuation (RMSF) for each residue (Figure 5). High RMSF values indicate higher fluctuations and flexibility. The average RMSF value was 1.43. However, there are two regions where the RMSF was higher than 3.0 Å. The first region (residues between Phe129 and His134) is centered around Asp131, with a maximum RMSF of 4.68 Å. It includes residues from β and γ turns and one residue from the H5 α-helix (Figure S2). The second region (Val227-Met233) includes Ile230, the most flexible residue, with the RMSF being 8.34 Å. Val227 is the last residue of the H9 α-helix, and Ser228 and Gly229 form a β turn, while other residues are part of an unstructured loop. The last few residues on the *C*-terminus have higher than average RMSF values. Despite the observed flexibility of the protein, its secondary structure was well conserved throughout the simulation. The secondary structure along the trajectory was calculated using the DSSP algorithm, which assigns secondary structure motifs based on backbone amide and carbonyl atom positions [26]. The results supporting our conclusions are presented in Figure S3.

**Figure 6:**
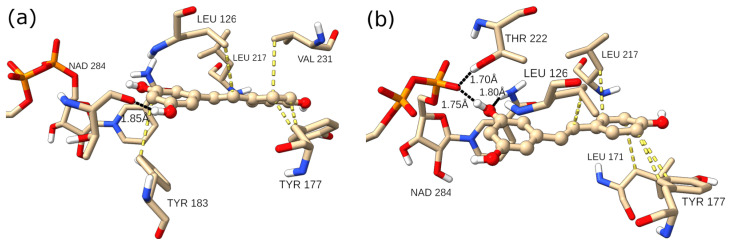
RES (ball and stick) in the catalytic pocket of 11β-HSD-1 for conformations (**a**) (left) and (**b**) (right) with key intermolecular interactions. Black lines indicate hydrogen bonds, while yellow lines are hydrophobic interactions.

RES is a small molecule (molecular weight 228.25 Da), obeying Lipinski’s rule of five. It has only two rotatable bonds, and with three hydroxy groups, it is able to form multiple hydrogen bonds, both as the hydrogen donor and as the hydrogen acceptor. Although the 11β-HSD-1 catalytic pocket is hidden from the solvent and is predominantly formed from hydrophobic amino acids, there are several residues with side chains capable of forming hydrogen bridges. The time evolution of the number of hydrogen bonds (H-bonds) between the RES ligand and the 11β-HSD-1 receptor is displayed in Figure 5 (right). The number of H-bonds varied between 0 and 5, and the theoretical maximal number of H-bonds (6) was not reached. On average, only 1.2 H-bonds were established. Closer analysis of representative structures of two clusters representing two dominant conformations A and B (vide infra) showed that the interaction pattern changed (Figure 6). For example, for conformation B, the one dominant in the first part of the simulation, two H-bonds with NAPDH cofactor were formed. Leu126, Tyr177, Tyr183, Leu217, and Val 231 are residues in the vicinity of RES, satisfying the 4.0 Å distance criterion between heavy atoms of RES and the residues. They all contribute to the stability of the RES:11β-HSD-1 complex by hydrophobic interactions. The key feature distinguishing conformation A’s ligand–receptor interaction map from conformation B’s is the reorganization of the H-bonds. While in B, NADPH acts both as a H donor and H acceptor to the same hydroxy group on the benzene-1,3-diol moiety of RES, NADPH does not form H-bonds at all with RES in A. In conformation A, NAPDH establishes only van der Waals contacts with RES. On the other hand, RES as a hydrogen donor forms a H-bond with Thr124 carbonyl oxygen, and the OH···O distance is 2.77 Å. It is interesting to see the dynamics of two hydrogen bonds that were observed in the highest number of frames during the simulation. The H-bond between the oxygen atom from NADPH and the hydroxyl group of RES existed for approximately 14% of the simulation time. The mean O···HO distance was 1.72 ± 0.14 Å, and it is one of the features of the first 200 ns of the dynamics. Between 200 and 400 ns, the distance varied around 6 Å, with an abrupt increase to ~9 Å. After that period, a new H-bond was formed, involving the same RES OH group and Thr124 as a hydrogen acceptor. This bond was present in 59% of the frames, with an average distance of 1.77 ± 0.12 Å (Figure S4).

### 2.3. Identification of Three Conformations

**Figure 7:**
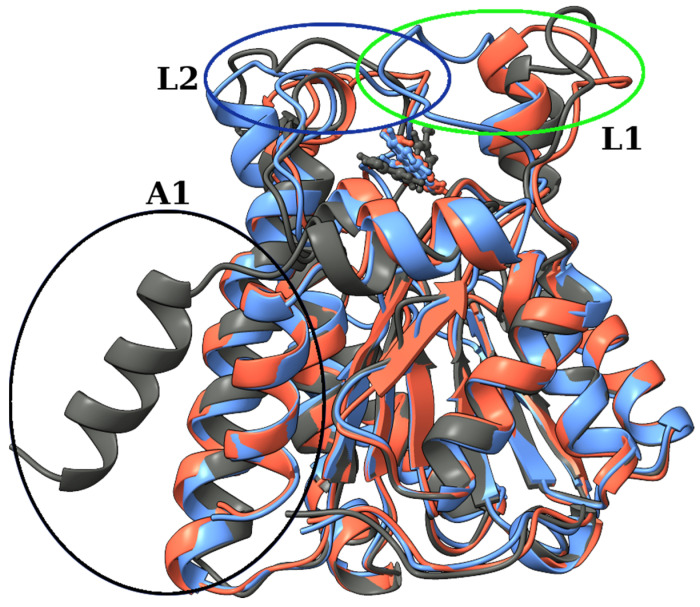
Overlay of three structures of RES:11β-HSD-1 complex representing three conformations, with encircled areas showing major structural rearrangements. Conformations A (blue), B (red), and C (gray), with encircled areas showing major structural rearrangements (A1, L1, and L2).

We performed a *k*-means cluster analysis based on the RMSD of Cα atoms to identify different conformations of 11β-HSD-1. The algorithm was set to have *k* between 2 and 10, and the results were analyzed using the Davies–Bouldin index (DBI), the pseudo-F statistic (pSF), and the ratio of sum of squares regression and sum of squares error (SSR/SST) (Table S1). The analysis confirmed the existence of three relevant conformations with relevant parameters collected in Table 3. For each conformation, the representative structure was obtained as the frame closest to the cluster’s centroid. In Figure 7***,*** the representative structures of all three conformations are compared. The RMSD relative to the first structure from the MD simulation with the position of representative conformations is shown in Figure 4. Conformation C is present only at the beginning of the trajectory. Within 4 ns, a large amplitude motion of the α-helix (H12; for notation, see Figure S2) at the *C*-terminus side of the protein (A1 on Figure 7) is observed. It moves closer to the H1 and H8 helices. These changes are reflected by an increase in RMSD (inset in Figure 4) and lowering of the values of the radius of gyration, indicating that the complex becomes more compact. Around 300 ns, a conformational change from B to A occurred. The two conformations differ in the position of the 10-residue-long unstructured loop between H9 and H10 α-helices (L1 on Figure 7). Residues Leu128 to Asp132 in A moved closer to the H8 helix compared to B, causing simultaneous movement of the upper part of the H5 helix (L1 on Figure 7). The residues that were responsible for this change turned out to have RMSF values higher than 3 Å (Figure 5). Since both changes were in the vicinity of the active pocket, the changes that the catalytic pocket experienced are serious (Figure S5). To describe the geometry of the protein pocket, our analysis clearly demonstrated the existence of two forms of the 11β-HSD-1 enzyme: an ‘open’ form (conformation B and C) and a ‘closed’ form (conformation A). The reorganization of the L1 loop encloses the ligand (RES) entirely, completely blocking access to the solvent molecules.

To gain additional insight into the conformational changes, a dynamics cross-correlation map (DCCM) and principal component (PC) analysis were performed. The cross-correlated values vary between 1 and −1. Negative values represent anti-correlated motions and positive values represent positively correlated motions. Residues from Pro29 to Met110 experienced correlated motion (Figure S6), and the mostly negative values of the off-diagonal elements of the dynamics cross-correlation matrix with other residues indicate anti-correlated movements. As expected, motion of Val227-Met233 (L1 region) is highly correlated. It also shows a positive correlation with residues from the L2 region and a neutral to slightly negative correlation with the motion of the H12 helix from the A1 region.

Coordinates of Cα atoms were used to construct a covariance matrix. PCA, a dimensionality reduction method, was performed to reduce the complexity of the dynamics and to extract the structural variations and collective motions of the enzyme. Diagonalization of the atomic fluctuation covariance matrix resulted in 789 eigenvectors with associated eigenvalues. The eigenvectors with the largest eigenvalues correspond to the most relevant atomic motions. By projecting Cα atoms’ displacements onto the obtained eigenvectors, we obtained principal components (PCs). In Figure S7, projections of the displacements of Cα atoms along PC1 and PC2 during MD simulation are presented, with additional information about the conformations. The first two components (PC1 and PC2) capture almost 60% and the first ten components capture more than 77% of the overall fluctuations. Visualization of the first two principal modes (Figure S8) corroborates observations and conclusions discussed earlier, pointing to the largest fluctuations and large amplitude motions of the L1, L2, and A1 regions.

### 2.4. Binding Free Energy Calculation

The RES binding affinity was estimated by the MM/GBSA approach, where, by solving the generalized Born equation, the solvation free energies were obtained. The final binding free energy is the sum of van der Waals, electrostatic, polar, and non-polar contributions (formulas (1)–(3)), plus entropic contribution at a temperature of 310 K. For the RES:11β-HSD-1 complex, the binding energy (without the unfavorable entropy contribution) was −23.6 ± 2.1 kcal mol^−1^. As mentioned in the Methods section, due to the high computational cost of evaluation of the TΔS part of the equation, only five structures per 50 ns sets were taken into consideration to calculate TΔS. The mean entropy multiplied by 310 K was −19.0 ± 1.9 kcal mol^−1^, making the final binding free energy −4.6 kcal mol^−1^.

**Figure 8:**
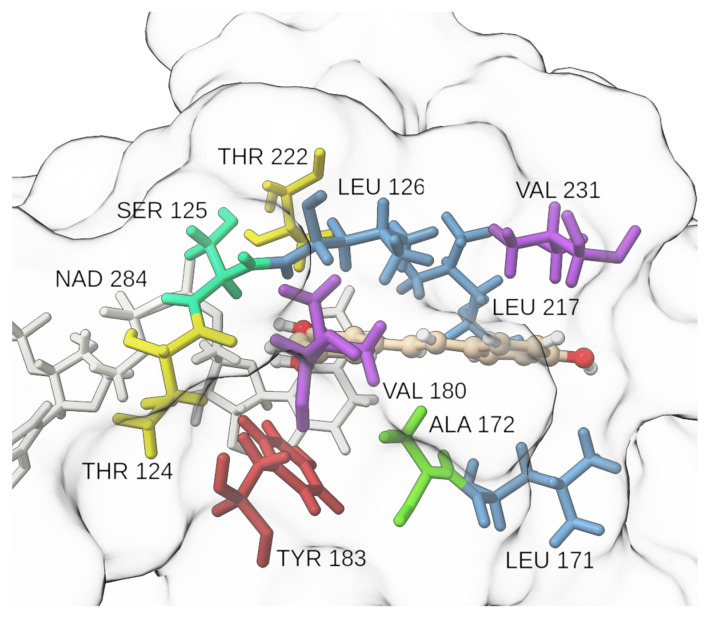
Insight into the catalytic site neighborhood for the RES:11β-HSD-1 complex. Ala (green), Leu (blue), Thr (yellow), Tyr (red), Val (violet), white (NADPH), ball-and-stick model (RES).

We identified key residues with dominant contributions to the protein–ligand binding, applying the MM/GBSA binding free energy decomposition. In our previous research on the SARS-CoV-2 virus main protease [27], Kyasanur forest disease virus NS3 protease [28], and in the development of potent pyrimidine–sulfonamide hybrids as selective BRAFV600E inhibitors [29], a threshold of −1.5 kcal mol^−1^ was set for a single residue binding free energy to classify it as a residue with a dominant contribution, and the same criteria were applied in the present study. Only three residues satisfied the criteria, namely Leu126 (−2.2 kcal mol^−1^), Leu217 (−1.7 kcal mol^−1^), and Leu171 (−1.6 kcal mol^−1^) (Figure 8). This observation supports other findings from the present study that hydrophobic interactions play an important role in the binding of RES into the active site of 11β-HSD-1. Thr124, the residue forming a H-bond with the ligand in conformation A, contributes with −1.2 kcal mol^−1^ to the binding free energy. Tyr183′s hydroxyphenyl moiety encloses a 42° angle with RES’s phenyl ring in the representative structure of conformation A and is capable of forming aromatic interactions.

## 3. Discussion

Our initial hypotheses were the following: (A) the ability of resveratrol to exert a protective effect against PTSD is related to its inhibitory effect on 11β-HSD-1 activity. Our own results, as well as results from literary sources demonstrating the key role of 11β-HSD-1 in the pathogenesis of PTSD, supported this hypothesis. (B) According to Tagawa et al. [30], resveratrol inhibits 11β-HSD-1 activity in vitro. These results were obtained on rat hepatocyte and adipocyte microsomes, indicating their non-specific nature. Therefore, we considered the inhibitory effect of resveratrol on 11β-HSD-1 activity to be fully established, relying on literature sources on this subject. Two different directions of our study pointed to the same conclusion. First, we demonstrated for the first time that resveratrol inhibits 11β-HSD-1 activity not only in vitro but also in vivo.

Second, in silico simulations were used to explain the nature of the inhibitory effect of resveratrol. According to Tagawa et al. [30], resveratrol has been shown to inhibit 11β-HSD-1 activity by a non-competitive mechanism. We identified resveratrol binding sites and showed that they are located outside the active site.

The in silico studies showed that resveratrol is able to bind to 11β-HSD-1, mainly via hydrophobic interactions. Other relevant interactions in the most populated conformation (A) included H-bonding between the Thr124 residue and RES and interactions of the hydroxyphenyl fragment of Tyr183 with the phenyl ring of RES. The results revealed a possible mechanism for resveratrol to have a direct effect on the metabolism of glucocorticoids in the liver. It remains unknown how the binding of resveratrol to 11β-HSD-1 affects its enzymatic activity. Currently, data are available demonstrating the ability of resveratrol to inhibit the activity of the enzyme after incubation with microsomes [30]. Therefore, resveratrol can be assumed to inhibit the activity of the enzyme by directly binding to it. This assumption is supported by our in vivo data. Resveratrol at a dose of 40 mg/kg suppressed 11β-HSD-1 activity in the liver of both unstressed and stressed rats. Moreover, the decreased activity of 11β-HSD-1 in unstressed rats was accompanied by a concomitant increase in corticosterone levels compared with controls, which is in agreement with data demonstrating a significant role of this enzyme in regulating blood corticosterone levels. It is noteworthy that resveratrol at a dose of 40 mg/kg also led to a reduction in anxiety levels compared to the non-stressed animals. In this context, it should be noted that there is a wealth of data demonstrating the protective effects of resveratrol in relation to behavioral activity.

Of particular interest here are the data comparing the behavioral effects of resveratrol with its effect on levels of monoamine neurotransmitters. Specifically, resveratrol at a dose of 80 mg/kg was found to effectively correct chronic stress-induced disturbances in the metabolism of monoamine neurotransmitters, as manifested by blocking a decrease in the levels of serotonin and norepinephrine in the hippocampus and hypothalamus [31]. During chronic stress, the decrease in serotonin and norepinephrine levels in these regions is accompanied by an increase in monoamine oxidase-A (MAO-A) activity [32]. This enzyme is involved in the regulation of the content of monoamine neurotransmitters in the brain through the reaction of their oxidative deamination. Resveratrol at a dose of 80 mg/kg reduced the activity of MAO-A in the hippocampus and hypothalamus in stressed animals [31]. Glucocorticoids, in turn, regulate the activity and expression of MAO–A [33]. Therefore, these stress hormones may indirectly affect the concentration of monoamine neurotransmitters in brain structures by regulating MAO-A activity. In studying the dynamics of experimental PTSD in rats, we found a correlation between 11β-HSD-1 activation in the liver and the development of anxiety [24]. Subsequently, a mathematical model was developed, represented by a system of differential equations, which defined the underlying biochemical variables and predicted the factors affecting anxiety development and dynamics. In the model, hepatic 11β-HSD-1 was regarded as a key determinant of plasma corticosteroid dynamics. In turn, plasma corticosterone controlled the dynamics of MAO-A activity in the brain, and MAO-A activity controlled the dynamics of norepinephrine in the brain. In this case, plasma corticosterone was represented as a determinant of anxiety. This mathematical model was in agreement with our own experimental data as well as with literature data showing resistance to PTSD in mice in which 11β-HSD-1 was genetically knocked out [25].

Based on the presented mathematical model, it is possible to understand how the observed effects of resveratrol on hepatic 11β-HSD-1 contribute to limiting anxiety in experimental PTSD. It is noteworthy that even administration of a dose of 40 mg/kg to non-stressed rats was accompanied by a decrease in hepatic 11β-HSD-1 activity with a concomitant decrease in anxiety levels according to the plus maze test. In stressed animals, administration of this dose resulted in similar changes in hepatic 11β-HSD-1 activity and behavioral activity. At the same time, the presence of strong positive correlations between 11β-HSD-1 activity and anxiety index in both unstressed and stressed rats draws attention. It is likely that these correlations reflect the significant contribution of this enzyme in the maintenance of blood corticosterone levels, which in turn influences the activity of MAO-A and the level of norepinephrine. As shown by our preliminary results, under conditions of experimental PTSD, increased activity of 11β-HSD-1 is accompanied by decreased blood corticosterone levels, resulting in decreased activity of MAO-A and an increased concentration of norepinephrine in the brain [24]. It is known that sustained activation of the noradrenergic system leads to the development of anxiety disorders.

## 4. Materials and Methods

### 4.1. Molecular Docking

The high-resolution (1.96 Å) 3D structure of 11β-hydroxysteroid dehydrogenase type 1 (11β-HSD-1) was obtained from the RCSB protein database [34]. In the catalytic pocket of the enzyme (PDB ID: 4K1L), a ligand SFF ((4aS,8aR)-*N*-cyclohexyl-4a,5,6,7,8,8a-hexahydro-4,1,2-benzoxathiazin-3-amine 1,1-dioxide) was bound. Only the protein’s A chain and NADPH were retained, while water and other small molecules were removed. The initial geometry of the resveratrol–11β-HSD-1 (RES:11β-HSD-1) complex was obtained by molecular docking simulation. The geometry of resveratrol (RES) was downloaded from PubChem [35]. The AutoDockTools 4 Python script prepare_ligand4.py [36] was used to prepare resveratrol and SFF. SFF was docked to the receptor with the aim of validating our docking procedure. The 11β-HSD-1 was prepared using Chimera 1.14 [37]. This involved Gasteiger charges being added to each atom and the merging of all non-polar hydrogens. Atom types were then ascertained, and the structure of the prepared receptor was saved as a pdbqt file. The center of the grid box was set to the SFF N13 atom, with Cartesian coordinates of −8.4, −1.1, and 40.6. The size of the box was 25 × 25 × 25 Å^3^, and both the exhaustiveness and the number of modes were set to 100. To save docked conformations, a 4 kcal mol^−1^ threshold relative to the highest score was established. After visual inspection of the results, the conformation with the lowest binding energy was kept. AutoDock Vina [36] was used for molecular docking simulations.

### 4.2. Molecular Dynamics Simulations

The initial structure of the RES:11β-HSD-1 complex for MD simulation was obtained by a molecular docking experiment. Parametrization of resveratrol was performed using the AMBER 20 Antechamber module [38] and the GAFF [39] force field. For the protein, the AMBER ff19SB [40] force field was employed. The protonation state of residues’ side chains was determined by the PDB2PQR web server [41]. The complex was soaked in a truncated octahedral periodic box of TIP3P water molecules. The minimum distance from the edges of the water box to the closest atom of the RES:11β-HSD-1 complex was set to 12 Å. The system was neutralized with the addition of four Na^+^ cations; then, as recommended by Machado and Pantano [42], Na^+^ and Cl^−^ ions were added to produce a neutral environment with a salt concentration of 0.15 M.

The following protocol for minimization–heating–equilibration–production was used. Firstly, while using periodic boundary conditions in all directions, the system was submitted for geometry optimization. In 10,000 optimization cycles (6000 conjugate gradient + 4000 steepest descent), both the ligand and the protein were restrained with harmonic potential *k* = 10.0 mol^−1^ Å^−2^. The system was gradually heated from 0 K to 310 K in 500 ps with no restrictions. After a 500-picosecond equilibration phase, a productive unconstrained molecular dynamics (MD) simulation of 1 μs was started. A time step of 2 fs at constant pressure (1 atm) and constant temperature (310 K) was employed. For temperature control, a Langevin thermostat with a collision frequency of 1 ps^−1^ was used. In all simulation protocols, hydrogen atoms were constrained by the SHAKE algorithm [43]. The exclusion criterion for non-bonded interaction was a distance of 11 Å, while long-range electrostatic interactions were treated with the particle mesh Ewald method [44]. In all directions, periodic boundary conditions were used. MD simulations were performed using the Amber molecular dynamics package [45]. MD simulations were performed on the Isabella cluster of the University Computing Center of the University of Zagreb, Croatia.

### 4.3. Binding Free Energy Calculation

The binding free energy (Δ*G_bind_*) between RES and 11β-HSD-1 was calculated according to the well-established molecular mechanics/generalized Born surface area (MM/GBSA) protocol [46]. The following formulae within a single-trajectory approach were implemented in the MMPSBA.py script of AmberTools’ package:

(1) $$∆G_{bind}=∆H-T∆S\approx ∆E_{MM}+∆G_{sol}-T∆S$$

(2) $$∆E_{MM}=∆E_{internal}+∆E_{\mathrm{electrostatic}}+∆E_{vdW}$$

(3) $$∆G_{sol}=∆G_{GB}+∆G_{SA}$$

where $∆E_{MM}$, the change in the MM energy contribution in the gas phase, is a sum of the bond, angle and dihedral energy ($∆E_{internal}$), and electrostatic ($∆E_{\mathrm{electrostatic}}$) and van der Waals ($∆E_{vdW}$) energies; $∆G_{sol}$ is the change in the solvation free energy, composed of the polar component ($∆G_{GB}$, electrostatic solvation energy) and non-polar, non-electrostatic solvation contribution ($∆G_{SA}$); and −T∆S is the conformational entropy upon binding. The production phase trajectory was divided into 20 parts of 50 ns length. From each segment, 100 snapshots were sampled at regular time steps and $∆G_{bind}$ was calculated. The final $∆G_{{bind}^{d}}$ was reported as the mean ± standard deviation of all 20 parts. The calculated MM/GBSA binding free energies were further broken down on a per-residue basis into specific residue contribution. In this way, the impact of each amino acid side chain on $∆G_{bind}$ was obtained and the character of the energy change in terms of entropic contributions or solvation energies and interaction was identified [47]. Due to the high computational cost of entropy contribution calculations, the entropy term was estimated based on 5 snapshots per 50 ns set, and the reported value is an average over 20 sets.

### 4.4. Dynamics Cross-Correlation Map (DCCM) Analysis

The degree of correlated atomic motions of the RES:11β-HSD-1 complex residues was computed using the DCCM approach [48,49] as implemented in Amber’s pytraj trajectory analysis module [50]. Only the Cα atomic coordinates of each amino acid were considered to reduce the statistical noise. The elements *C_ij_* of the covariance matrix were calculated as follows:

(4) $$C_{ij}=\frac{\langle r_{i}r_{j}\rangle -\langle r_{i}\rangle \langle r_{j}\rangle }{{\left[\left(\langle r_{i}^{2}\rangle -\langle r_{i}\rangle {}^{2})(\langle r_{j}^{2}\rangle -\langle r_{j}\rangle {}^{2}\right)\right]}^{1/2}}$$

where **r***_i_* and **r***_j_* are the position vectors of two Cα atoms *i* and *j*, respectively. Angle brackets denote time averages over the entire trajectory.

### 4.5. Cluster Analysis

Using the *k*-means algorithm, RES:11β-HSD-1 structures were sorted into three clusters based on the RMSD of Cα atoms of each residue. The maximal number of iterations was set to 1000, with a randomized original set of points and sieving set equal to 10. The frames closest to the clusters’ centroids were distinguished and were considered as structures characteristic of three conformations. The CPPTRAJ module [50] was used to perform cluster analysis.

### 4.6. Principal Component Analysis (PCA)

Prior to PCA, the trajectory was fitted on an average structure. Only then was the covariance matrix calculated. Atomic fluctuations of Cα atoms of each residue were used to construct a covariance matrix. Its diagonalization provided a set of orthogonal eigenvectors with respective eigenvalues. PCA was performed using the scikit-learn library [51] in Python 3.6.

### 4.7. In Vivo Experiments

#### 4.7.1. Protocol No. 1

The study was performed on 36 male Wistar rats weighing 245–280 g. These studies tested the effects of resveratrol at doses of 10, 25, and 40 mg/kg, administered intragastrically for 10 days, on liver 11β-HSD-1 activity, plasma corticosterone concentrations, and measures of behavioral activity in the elevated plus maze (EPM) test (i.e., elevated plus-shaped maze). *Trans*-resveratrol was purchased from Sigma Aldrich Ltd. (Sent Louis, MO, USA) and dissolved in a 0.5% sodium carboxymethylcellulose solution for oral administration via the gastrointestinal tract.

The choice of this dose was based on Li et al. [52], who demonstrated the efficacy of the aforementioned resveratrol administration in relation to experimental PTSD. Control rats were orally administered a 0.5% sodium carboxymethylcellulose solution (Carbocell, Russian Federation, Ekaterinburg) by gavage. As a first step, we determined the most effective dose in terms of hepatic 11β-HSD-1 activity to test its effect on experimental PTSD. The following groups were introduced in this experiment:

1. Control rats (treated with vehicle only for 10 days; *n* = 10);
2. RES1 (treatment with a dose of 10 mg/kg for 10 days; *n* = 6);
3. RES2 (treatment with a dose of 25 mg/kg for 10 days; *n* = 10);
4. RES3 (treatment with a dose of 40 mg/kg for 10 days; *n* = 10).

Treatment with resveratrol was performed for 10 days. On day 11, an EPM test was performed and the animals were sacrificed. Rats were sacrificed with an overdose of diethyl ether and decapitated, and blood was collected. Blood plasma and liver were kept frozen at −70 °C for biochemical studies.

#### 4.7.2. Protocol No. 2

The study was performed on 30 male Wistar rats weighing 230–270 g. The animals were divided into the following groups:

1. Control rats (treated with vehicle only for 10 days; *n* = 10)
2. PTSD (rats previously exposed to chronic predator stress followed by a two-week break; *n* = 10)
3. RES + PTSD (an effective dose of resveratrol administered to rats via a tube one hour before the onset of predatory stress; *n* = 10). The effective dose of resveratrol was determined after protocol № 1 was performed.

To induce PTSD, we used a modified predator stress model originally described by Cohen and Zohar [53], as was used in our previous studies [54]. Predator stress was achieved by exposing rats in the PTSD groups to the smell of cat urine for 15 min daily for 10 days. Subsequently, PTSD rats were given a 14-day rest period under stress-free conditions. On day 15, an EPM test was performed and the animals were sacrificed. The rats were sacrificed with an overdose of diethyl ether and decapitated, and the blood was collected. Blood plasma and liver were kept frozen at −70 °C for biochemical studies.

#### 4.7.3. Behavioral Assessment

On day 14 after the last exposure to PSS, the anxiety level of the rats was measured using the elevated plus maze (EPM) test, as described previously. The test lasted for ten minutes. AI was calculated using the following formula:

(5) $$AI=1-\frac{1}{2}\left(\frac{T_{op}}{T}+\frac{N_{op}}{N}\right)$$

where *T_op_* is the time spent in the open arms, *T* is the total time in the maze, *N_op_* is the number of entrances to the open arms, and *N* is the total number of all entrances. The elevated plus maze (EPM) test is one of the most commonly used tests to investigate anxiety-like behavior in rodents. The apparatus consisted of four branched arms (50 × 10 cm) with two open arms and two closed arms (40 cm high). The arms were connected by a central arm.

Plasma concentrations of CORT were determined using an enzyme-linked immunosorbent assay (ELISA) kit for measuring CORT (Cusabio ELISA Kit, Houston, TX, USA) according to the manufacturer’s instructions. The sensitivity of the assay was 0.25 ng/mL, and the coefficients of variation within and between assays were <5%.

Hepatic 11-βHSD-1 activity was evaluated by a decrease of 10 μM corticosterone (Sigma Aldrich Ltd., Sent Louis, Missury, MO, USA). A total of 0.1 M sodium phosphate buffer (pH 8.5) containing 1.5 mM NADP (Sigma Aldrich Ltd., Sent Louis, MO, USA) was used. Incubation of the samples was conducted for 60 min at 37 °C. The sample containing the substrate (corticosterone) was added after the end of incubation, and the blank sample containing an equivalent volume of solvent was incubated simultaneously. The changes in fluorescence intensity (405 nm excitation wavelength and 546 nm emission wavelength) were measured using a VERSA FLUOR spectrofluorometer (Bio-Rad, Hercules, CA, USA).

#### 4.7.4. Statistical Analysis

Data were analyzed using SPSS 24.0 (SPSS Inc., Chicago, IL, USA), STATISTICA 10.0 (StstSoft Inc., USA), and MS Excel 2010 (Microsoft Inc., Redmond, DC, USA) software. Quantitative data are presented as mean ± SD. After the Shapiro–Wilk test showed normal distribution, a one-sided ANOVA was performed with Tukey’s post hoc tests.

## 5. Conclusions

The results of the in silico and in vivo studies can be summarized as follows. The in silico data indicate the ability of resveratrol to interact directly with the 11β-HSD-1 protein through hydrogen bonding and hydrophobic interactions. The in vivo data suggest that resveratrol at a dose of 40 mg/kg causes a decrease in 11β-HSD-1 activity in the liver of non-stressed animals. The same dose of resveratrol effectively prevented the increase in 11β-HSD-1 activity in the liver of stressed individuals and prevented the development of anxiety disorders in experimental PTSD. Thus, the ability of resveratrol to limit behavioral disturbances in PTSD is related to its effect on 11β-HSD-1-dependent tissue metabolism of glucocorticoids in the liver.

## Figures and Tables

**Table 1:** Effect of RES treatment on EPM test scores.

Variable	Control	RES1 (10 mg/kg)	RES2 (25 mg/kg)	RES3 (40 mg/kg)
*N*	10	6	10	10
Time spent in closed arms/s	478.7 ± 28.37	471.5 ± 30.11	495 ± 29.59	342.1 ± 108.72 **
Time spent in closed arms/s	121.3 ± 28.37	128.5 ± 30.11	105 ± 29.59	257.9 ± 108.72 **
Entries into closed arms	9.4 ± 3.5	6.6 ± 1.86	12.5 ± 5.89	4.6 ± 2.5 **
Entries into open arms	5.7 ± 1.6	4.5 ± 1.22	6.6 ± 3.65	5.8 ± 2.9
AI	0.7 ± 0.08	0.71 ± 0.05	0.77 ± 0.09	0.51 ± 0.09 ***

** *p* < 0.01, *** *p* < 0.0001, respective control vs. RES3.

**Table 2:** Resveratrol treatment prevented the development of anxiety-like behavior in experimental PTSD rats.

Variable	Control	PTSD	PTSD + RES (40 mg/kg)
*n*	10	10	10
Time spent in closed arms/s	461.4 ± 37.4	554 ± 25.44 ***	451.4 ± 36.9 ^##^
Time spent in closed arms/s	138.5 ± 37.4	46 ± 25.44 ***	148.6 ± 36.9 ^##^
Entries into closed arms	7.00 ± 4	5.5 ± 3.3 *	13.3 ± 7.7 ^##^
Entries into open arms	4.1 ± 1.5	2.2 ± 1.9	8.3 ± 6.1 ^##^
AI	0.68 ± 0.09	0.83 ± 0.06 ***	0.69 ± 0.05 ^##^

* *p* < 0.05, *** *p* < 0.001 respective, control vs. PTSD; ^##^ *p* < 0.01 respective, PTSD vs. PTSD + RES.

**Table 3:** Cluster occupancy of RES:11β-HSD-1 complex through time (1 μs).

Cluster	Conformation	Cluster Population	d ^a^	csd ^b^	RMSD against A
1	A	0.705	2.253	0.251	0
2	B	0.291	2.524	0.425	2.858
3	C	0.004	1.857	0.236	3.816

^a^ d = average distance between points in the cluster; ^b^ csd = standard deviation of points in the cluster.

## Data Availability

Data are contained within the article and the supplementary materials.

## Supplementary Materials

The following supporting information can be downloaded at: https://www.mdpi.com/article/10.3390/ph16020251/s1, Figure S1: Overlay of experimental (green) and docked (red) SFF structures within the 11β-HSD-1 protein pocket; Figure S2: Secondary structure of the 11β-HSD-1 protein. Figure was generated with the PDBsum web server [55]; Figure S3: Changes in the secondary structure of the 11β-HSD-1 protein during molecular dynamics simulation; Figure S4: Time evolution of the two most populated hydrogen bonds between RES and 11β-HSD-1 during the MD simulation; Figure S5: Geometry of the catalytic pocket in two representative conformations A (left) and B (right); Figure S6: Dynamic cross-correlation heat map for the RES:11β-HSD-1 complex. Correlated movements are encoded by the red color, anti-correlated movements by the blue color; Figure S7: Two-dimensional PCA scatterplots showing the projections of the displacement of the Cα atoms along the first two eigenvectors for each frame of the RES:11β-HSD-1 complex. Conformations A (blue), B (red), and C (black); Figure S8: Visualization of the PC1 (left) and PC2 (right) modes of the RES:11β-HSD-1 complex; Table S1: Clustering statistics for the RES:11β-HSD-1 complex.
