# Supplementary material for: Can Resveratrol Influence the Activity of 11β-Hydroxysteroid Dehydrogenase Type 1? A Combined In Silico and In Vivo Study
Source: Pharmaceuticals (Basel). 2023 Feb 7;16(2):251. doi: 10.3390/ph16020251 (PMC9960807; doi:10.3390/ph16020251)
Supplement: Supplementary file 1 [file pharmaceuticals-16-00251-s001.zip › pharmaceuticals-2108342-supplementary.pdf]

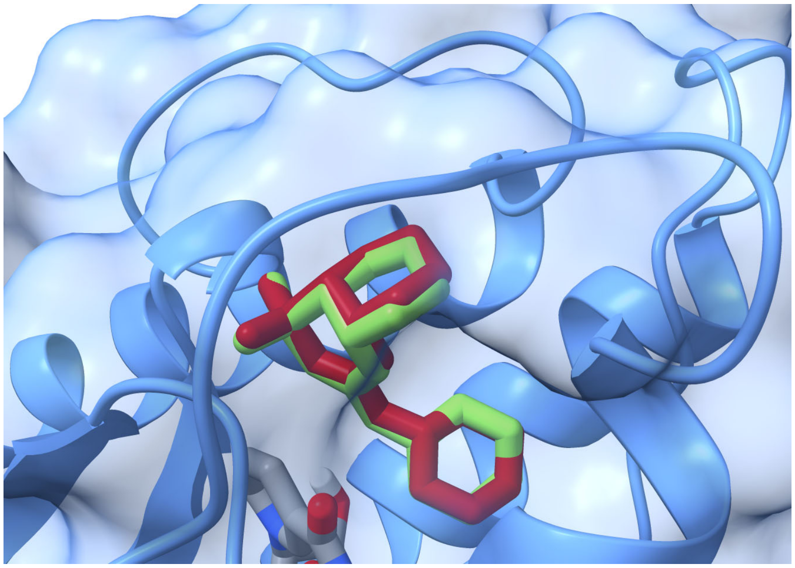

Figure S1. Overlay of experimental (green) and docked (red) SFF structures within the 11β-HSD-1 protein pocket.

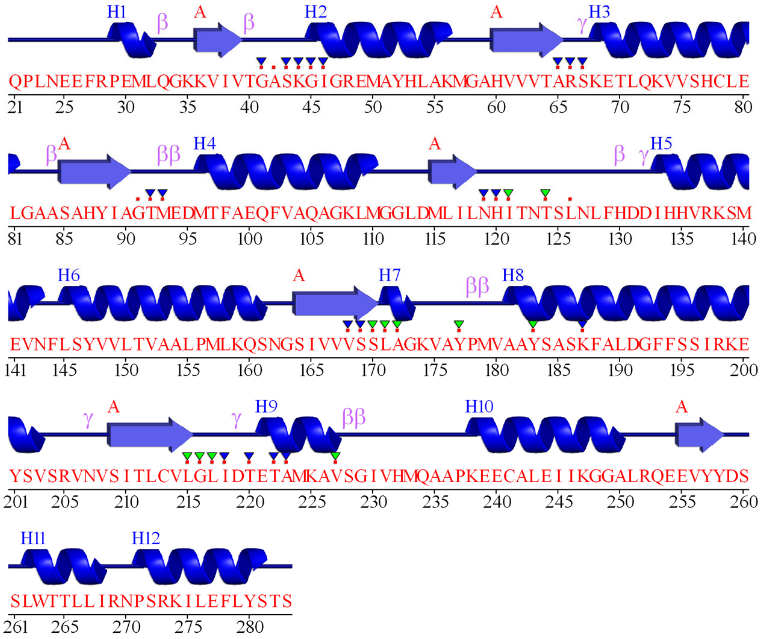

Figure S2. Secondary structure of the 11β-HSD-1 protein. Figure was generated with the PDBsum web server [55].

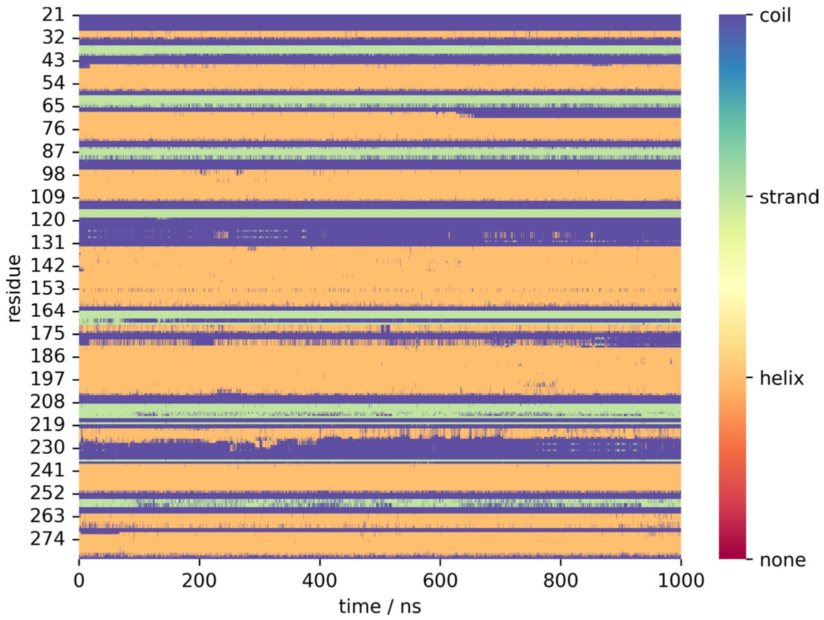

Figure S3. Changes in the secondary structure of the 11β-HSD-1 protein during molecular dynamics simulation.

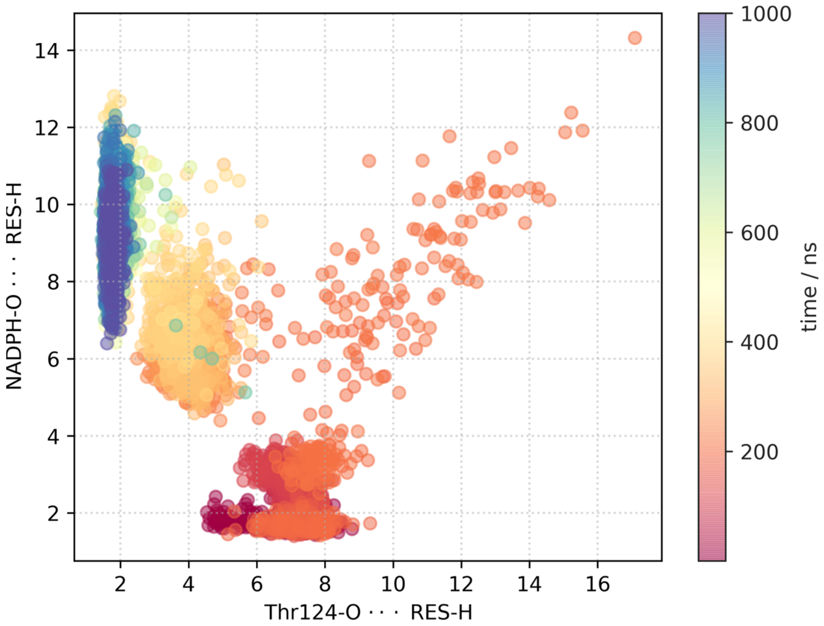

Figure S4. Time evolution of the two most populated hydrogen bonds between RES and 11β-HSD-1 during the MD simulation.

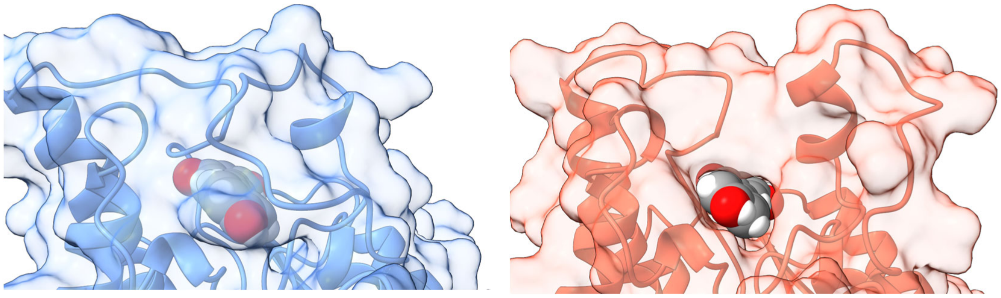

Figure S5. Geometry of the catalytic pocket in two representative conformations A (left) and B (right).

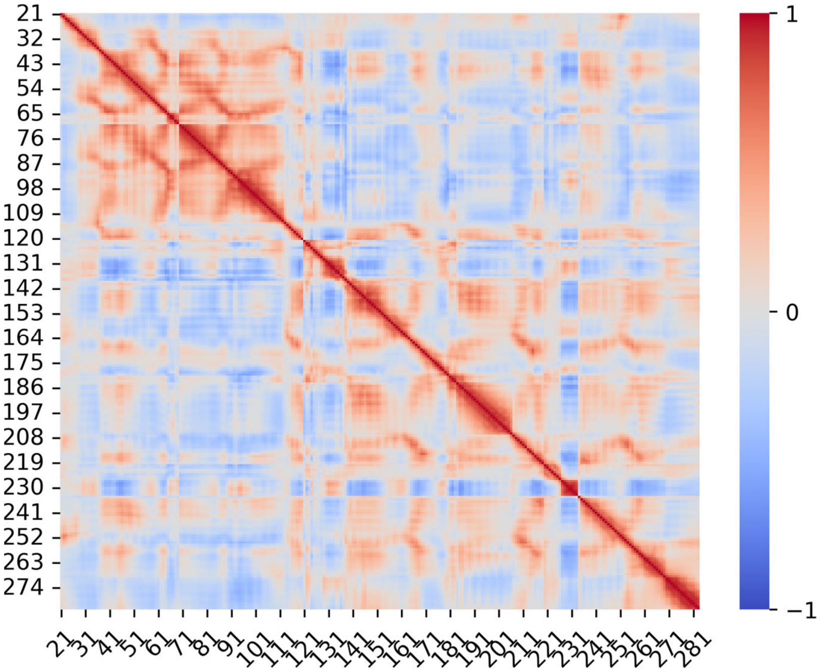

Figure S6. Dynamic cross-correlation heat map for the RES:11β-HSD-1 complex. Correlated movements are encoded by the red color, anti-correlated movements by the blue color.

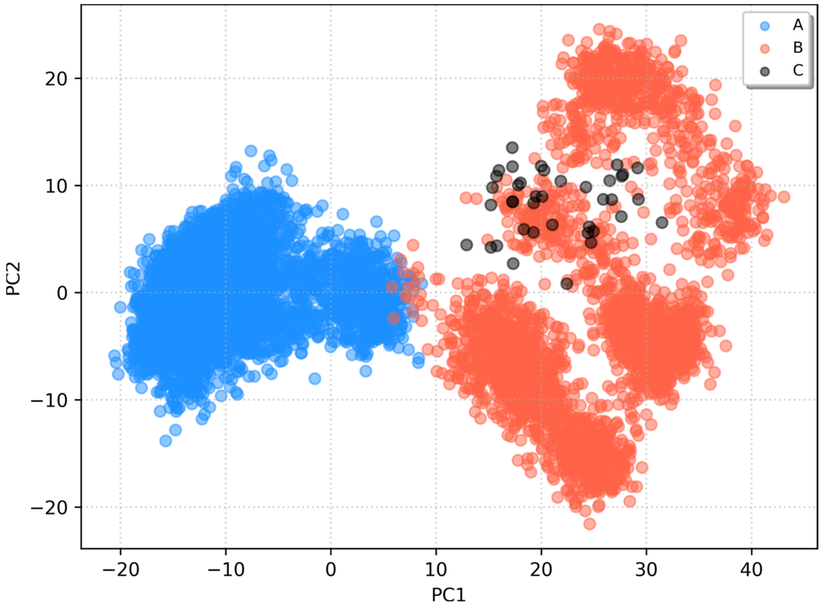

Figure S7. Two-dimensional PCA scatterplots showing the projections of the displacement of the Cα atoms along the first two eigenvectors for each frame of the RES:11β-HSD-1 complex. Conformations A (blue), B (red), and C (black).

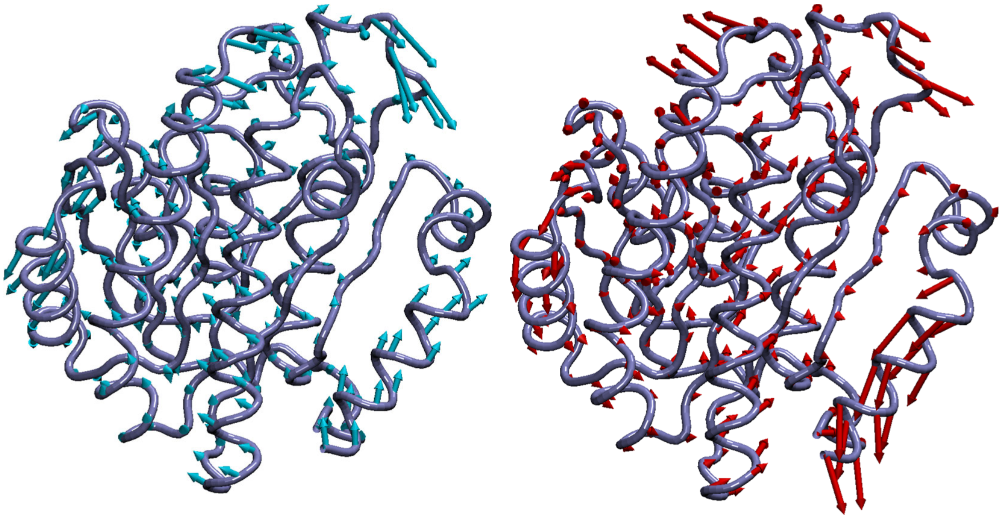

Figure S8. Visualization of the PC1 (left) and PC2 (right) modes of the RES:11β-HSD-1 complex.

Table S1. Clustering statistics for the RES:11β-HSD-1 complex.

# of clusters	DBI [a]	pSF [b]	SSR/SST [c]
2	1.249	115.7	0.011
3	1.298	2256.7	0.311
4	2.177	1823.5	0.354
5	2.202	1631.1	0.395
6	2.108	1310.4	0.396
7	2.062	1236.0	0.426
8	2.125	1187.2	0.454
9	2.045	1087.0	0.465
10	2.046	975.2	0.468

[a] DBI = Davies-Bouldin index, [b] pSF = pseudo-F statistic, [c] SSR/SST = ratio of sum of squares regression and sum of squares error
